# Maillard Reaction Products in Gluten-Free Bread Made from Raw and Roasted Buckwheat Flour

**DOI:** 10.3390/molecules26051361

**Published:** 2021-03-04

**Authors:** Maria Barbara Różańska, Aleksander Siger, Artur Szwengiel, Krzysztof Dziedzic, Sylwia Mildner-Szkudlarz

**Affiliations:** 1 Department of Food Technology of Plant Origin, Poznań University of Life Sciences, 31 Wojska Polskiego St., 60-624 Poznań, Poland; artur.szwengiel@up.poznan.pl (A.S.); krzysztof.dziedzic@up.poznan.pl (K.D.); 2 Department of Biochemistry and Food Analysis, Poznań University of Life Sciences, 60-623 Poznań, Poland; aleksander.siger@up.poznan.pl

**Keywords:** Maillard reaction products, furosine, fluorescent intermediate compounds, buckwheat bread, phenolic compounds, antioxidant activity

## Abstract

The formation of Maillard reaction products (MRPs) in gluten-free bread made from roasted and raw buckwheat flour was examined. The levels of phenolic compounds such as flavonoids (catechin, naringenin, quercetin, rutin, and others) and phenolic acids (like 4-hydroxybenzoic, caffeic, dihydroxybenzoic, ferulic, gallic, syringic, vanillic, and *p*-coumaric) were measured using reversed-phase ultra-high performance liquid chromatography-electrospray ionization mass spectrometry (RP–UHPLC–ESI-MS). Early and advanced Maillard reaction products were analyzed using HPLC, whereas spectrofluorimetric analysis was used to determine the levels of fluorescent intermediate compounds (FIC). The total levels of phenolic compounds were higher in the case of buckwheat bread prepared from roasted buckwheat flour (156 and 140 µg/g of crumb and crust, respectively). Rutin, gallic acid, and catechin were the most abundant phenolic compounds detected in roasted buckwheat bread. The roasting process resulted in significantly lower radical scavenging capacities (ABTS) of the total phenolics and flavonoids in the buckwheat bread. Taking into consideration these Maillard reaction products, we observed a significant increase in FIC level in roasted buckwheat crumb and crust (at about 40%, and 38%, respectively). At the same time, the Nε-(carboxymethyl)lysine (CML) level did not change in roasted or raw buckwheat bread crumb, though in roasted buckwheat crust the concentration of CML increased by about 21%.

## 1. Introduction

Bread is one of the most popular cereal products in the world. In the last decade, an increase in interest in gluten-free (GF) products was observed. Numerous studies have being conducted to improve the quality of gluten-free (GF) bread [1] given its poor nutritional value (manifesting as insufficient levels of protein and the minerals Ca, Fe, Mg, and Zn) [2], poor aroma [3], and poor textural quality. Like other pseudocereals (e.g., amaranth, quinoa), buckwheat flour does not contain proteins with the ability to trigger the auto-immune response observed in celiac disease, and therefore can be used in the production of gluten-free bread [4]. Two types of buckwheat flour are commercially available with high variability in the composition of nutrients. The first is raw buckwheat flour, produced by using raw dehulled grains; the second is roasted buckwheat flour, which is obtained from buckwheat grains roasted at 130 °C under 5–6 bars pressure for 1 h, and then dehulled [5].

Raw buckwheat is usually processed into flour, but in Central and Eastern Europe the roasting process is also used to produce buckwheat groats (the whole grains sold as a food product). It is known that heat treatment of buckwheat grains alters their functional and nutritional properties and can modify their chemical composition, so different prohealth effects can be achieved by using different types of buckwheat groats and flour in food production. It should be highlighted that buckwheat is one of the best pseudocereal sources of phenolic compounds [4]. In raw and roasted common buckwheat (*Fagopyrum esculentum*), ferulic acid (8.94 and 5.78mg/kg), gallic acid (2.84 and 9.63 mg/kg), rutin (62.19 and 43.21mg/kg), *p*-coumaric acid (1.83 and 1.78mg/kg), and quercetin (26.95 and 25.02 mg/kg) have been found [6]. However, thermal processing of buckwheat leads to a notable reduction in its antioxidant capacity [6,7,8]. For example, Ma et al. [9] found that the total phenolic content of roasted buckwheat was statistically significantly higher than raw common buckwheat, but the opposite tendency was observed for the antioxidant activity of Tartary buckwheat.

Furthermore, the industrial thermal process can limit the negative effects of potential allergens and toxins in roasted groats and flours, which may also be the reason for the growing popularity of these raw materials [10]. Thermal processing causes the formation of end-products of Maillard reaction—melanoidins, which capable of increasing the antioxidative activity of food [11]. Notwithstanding, heat treatment during the roasting and baking process affects the formation of potentially harmful Maillard reaction compounds, such as acrylamide (ACR), hydroxymethylfurfural (HMF) furfural, and especially advanced glycation end products (AGE) e.g., Nε-(carboxymethyl)lysine) [10,12,13]. Numerous recent studies have been conducted on the antiglycation effect of polyphenolic compounds in the food matrices [13,14], but there is a lack of information about Maillard reaction products (MRPs) formation in gluten-free bread made from buckwheat. Moreover, current research on the effect of heat treatment on buckwheat product mainly involves chemical composition changes and the Maillard reaction in single buckwheat type products, including buckwheat groats (kasha) and buckwheat flour [7,8,9].

The aim of this study was to assess the formation of Maillard reaction products during the production of gluten-free bread from raw and roasted buckwheat flour.

## 2. Results and Discussion

Results on the concentration of phenolic compounds in buckwheat bread are summarized in Table 1. In the case of crust and crumb of roasted buckwheat bread, the total phenolic compound content was 158% and 187% higher, respectively, than in bread produced with raw buckwheat flour. These findings can be related to the fact that the steaming processing in the production of roasted buckwheat flour led to a transfer of water-soluble substances from buckwheat husk to the grain [15]. Moreover, the degradation of the buckwheat grain cell membrane during the heat treatment could enhance the extractability of phenolic compounds from the bound fraction [16]. It should be highlighted that quercetin concentrations in raw buckwheat bread in the crumb and crust were 42–46 times higher than in the crumb and crust of the roasted buckwheat bread. These results are in line with those of Bhinder et al., who found that roasting Tartary buckwheat flour led to a decrease in quercetin content [10]. Previous studies confirmed that the thermal stability of quercetin, which is the aglycon of rutin, was lower than that of rutin, the most thermostable polyphenol [9,10,17]. It is likely that the activity of the enzymes that break down rutin during the heat treatment of buckwheat grain was inhibited in the production of roasted flour [18,19]. Hence, the lack of rutin-degrading enzymes in the roasted buckwheat flour during bread making did not lead to a decrease in rutin content in roasted buckwheat bread; this is unlike the situation with raw buckwheat bread, where rutin was not detected. The other abundant compounds in the roasted buckwheat bread, besides rutin, were gallic acid, dihydroxybenzoic acid, and catechin. The use of roasted buckwheat flour contributed to a significant increase in these compounds, suggesting that these compounds were liberated from bonded forms during heat treatment. Moreover, there were no significant differences in concentrations of gallic acid, dihydroxybenzoic acid, or catechin between the roasted buckwheat crumb and crust (Table 1).

The antioxidant activity of raw and roasted buckwheat bread is given in Table 1. The parameters of the roasting process, particularly temperature and time, are known to affect the antioxidant activity of various food products. Interestingly, the roasting process led to a significant decrease (*p* < 0.05) in the antioxidant activity of buckwheat bread. Zieliński et al. showed a loss of antioxidant activity in common buckwheat (*Fagopyrum esculentum*) after roasting at 160 °C [7]. The results presented here are in agreement with those of Sensoy et al. [20], who found that roasting dark buckwheat flour at 200 °C for 10 min led to an increase in nonpolar and polar compounds, but decreased their antioxidant activity. Besides phenolics, other compounds such as proteins can contribute to the measured antioxidant activity of buckwheat products [21]. Furthermore, according to Horáková, the prooxidant properties of flavonoids depend on their concentration [22]. It is therefore likely that a high flavonoid content in roasted buckwheat bread will also involve a pro-oxidative effect. Some authors have proposed that phenolic compounds, as well as ascorbic acid, are able to participate in the Maillard reaction, leading to a decrease in their antioxidant activity [23]. According to the literature, it is suggested that the reduction in antioxidant activity due to the roasting process may also be due to the formation of MRPs with pro-oxidant properties during the early stages of browning [24].

Research in recent years has focused on the inhibitory effect of phenolic compounds on the formation of MRPs [25,26]. Moreover, the antiglycation activity of phenolic compounds is usually associated with their antioxidant efficiency in the following decreasing order: quercetin > gallic acid > catechin > ferulic acid [27].

Table 2 shows the MRPs formed in bread crumb and crust formulated with roasted and raw buckwheat flour. Due to the high proportions of fat (10%) and sugar (9%), along with the yeast, it is difficult to compare the level of MRPs in buckwheat samples to other bakery products.

Furosine (FUR), formed during acid hydrolysis of Amadori rearrangement products (ARPs) from lysine, has been proposed as an indicator of the early stage Maillard reaction. It has been reported that FUR is an unsuitable marker for the effect of heat treatment during baking, as it rapidly changes into advanced MRPs [28]. However, the FUR content of buckwheat bread crumb and crust could be useful for understanding the Maillard reaction mechanism in the model buckwheat bread. The levels of FUR were significantly reduced (~91%) in buckwheat roasted crumb and crust (6.34 and 8.73 mg/kg, respectively) compared to raw buckwheat bread (71.56 and 117.56 mg/kg for crumb and crust, respectively). This could be explained by the fact that the buckwheat roasting process favors the formation of more advanced, fluorescent, cross-linking MRPs in the bread. Similarly, an increase in intermediary and advanced MRPs (fluorescent intermediate compounds (FIC) and Nɛ-carboxymethyl lysine (CML) level) was reported by Wronkowska et al. [8], who looked at changes in the formation of MRPs induced by buckwheat groats roasted at 160 °C for 30, 40, and 50 min. Çelik and Gökmen did not detect FUR in whole and refined wheat bread crust-like samples heated at 200 ℃ for over 15 min, which confirms the instability of FUR under heat treatment [29]. It is worth noting that in this study FUR levels in raw buckwheat bread crust (Table 2) were higher than > 100 mg/kg. In contrast, Çelik and Gökmen found that, in the whole and refined wheat bread crust-like samples, FUR concentration was lower (< 100 mg/kg) [29]. Moreover, highly significant negative correlations were found between FUR content and *p*-coumaric (−0.97, *p* < 0.05), catechin (−0.94, *p* < 0.05), rutin, gallic, ferulic, vanillic, and syringic acid (−0.93, *p* < 0.05) concentrations in the crumb and crust, indicating that they might be involved in the protein protection (Table 3).

On the other hand, buckwheat bread samples formulated with roasted buckwheat flour showed a statistically significant increase in their levels of FIC. Surprisingly, there in the level of FIC were no statistically significant differences between the level of FIC in the crust and the crumb of the buckwheat bread prepared from the same kind of flour. This may suggest that different conditions during baking, such as temperature and water activity inside and on the surface of the dough, do not significantly affect the level of these intermediary MRPs [30].

The negative correlation coefficient (−0.89, *p* < 0.05) between FUR and FIC confirms that this nonenzymatic browning reaction in the bread samples tended towards the formation of intermediary and subsequently more advanced MRPs. This observation is in agreement with Carciochi et al. [31], who found significant increases in FIC level after roasting quinoa seeds, at 160 °C and 190 °C for 60 to 90 min. It can further be assumed that a high concentration of quercetin in raw buckwheat flour markedly contribute to suppressing FIC formation (−0.98, *p* < 0.05). This is in line with the study of Zhang et al. [26] who reported that quercetin most strongly inhibits fluorescent AGEs in a cookie model, followed by naringenin, rosmarinic acid, and epicatechin.

One valuable marker for monitoring the progress of the Maillard reaction in food is Nɛ-carboxymethyl lysine (CML). The use of roasted buckwheat flour did not affect the CML content in the crumb obtained from raw buckwheat flour (Table 2). However, it should be emphasized that the process of roasting buckwheat grains increased (21.42%) the CML content of the buckwheat bread crust. It is known that the temperature of the crust exceeds that of the crumb by over 100 °C [32]. Heat transmission during the baking process thus favors the formation of Maillard reaction products (MRPs) in the crust. Compared to the literature data, discrepancies can be seen between the CML content of bread prepared from buckwheat flour and the model wheat bread (49.71 mg/kg bread and 15.09 mg/kg crumb) [13], as well as the model sponge cake prepared using a different type of sugar (1.16–6.64 mg/kg model cake) [33]. Interestingly, no significant correlation was found between CML and phenolic compounds content in the bread crumb and crust, regardless of the type of flour used. It should be highlighted that flavonoids with the 3-OH and 5-OH groups in the A ring may have the ability to trap reactive dicarbonyl compounds and thus inhibit the formation of advanced glycation end products (AGE) [34]. Despite the fact that rutin is considered to have antioxidant properties, in this study we noted no inhibitory effects on the formation of AGE. Moreover, the inhibitory effect of phenolic compounds can not only be associated with a high concentration of rutin because other compounds could also be responsible for antagonistic or synergistic effects on Maillard reaction product formation [35]. Starowicz et al. [36] stated that supplementation with rutin of rye and buckwheat biscuits increased the formation of volatile compounds typical of the advanced stage of the Maillard reaction. It can thus be suggested that a high level of quercetin 3β-d-rutinoside (rutin) in the crumb and crust of roasted buckwheat bread can also be involved in the formation of increased concentrations of advanced MRPs in the bread samples. These findings agree with those of Yuan et al. [37], who reported that the type and concentration of natural antioxidants significantly affect the formation of Maillard reaction products. A higher concentration of phenolic compounds can also have a promoting effect on the formation pathway of advanced MRPs. Conversely, Przygodzka et al. [38] reported that the enrichment of rye and buckwheat ginger cakes formulated from rye flour and roasted dehulled buckwheat flour with 50 mg and 100 mg rutin, respectively, did not result in an increase in CML, i.e., 24.36 ug/g without rutin; 23–83 μg/g with 50 mg of rutin, and 22–96 μg/g with 100 mg of rutin addition.

**Figure 1:**
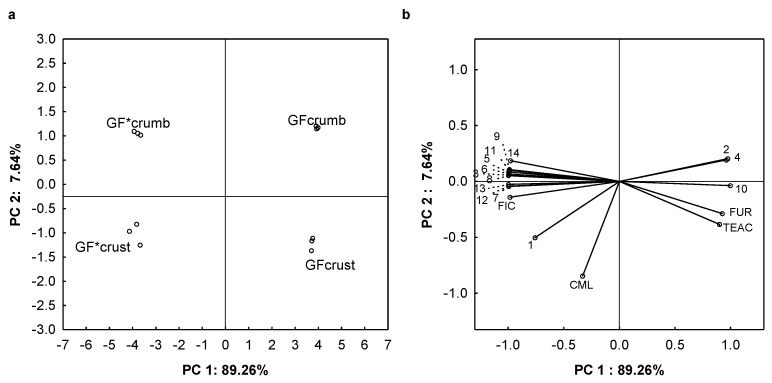
Principal component analysis (PCA) score plot (**a**): gluten-free (GF) crumb: GF bread crumb formulated with raw buckwheat flour; GF crust: GF bread crust formulated with raw buckwheat flour; GF*crumb: GF bread crumb formulated with roasted buckwheat flour; GF*crust: GF bread crust formulated with roasted buckwheat flour; loading plot (**b**): 1: 1-*O*-sinapoyl-beta-d-glucose; 2: 4-hydroxybenzoate-*O*-glucoside; 3: 4-hydroxybenzoic acid; 4: caffeic acid; 5: catechin, 6: dihydroxybenzoic acid; 7: ferulic acid; 8: gallic acid; 9: naringenin; 10: quercetin; 11: rutin; 12: syringic acid; 13: vanillic acid; 14: *p*-coumaric acid.

Principal component analysis (PCA) of the MRP and phenolic compound concentrations, as well as of the antioxidant activity of the samples was performed in order to identify the main factors determining the properties of the buckwheat bread (Figure 1). The two principal components (PCs) explained 96.93% of the total variance. The score plot (Figure 1a) shows a clear separation in the negative component of PC1 for the crumb and crust of the bread formulated with the roasted buckwheat flour, and in the positive scores of PC1 for the crumb and crust of the bread formulated with the raw buckwheat flour. This separation is related to the relative levels of FIC and CML the former being higher for the gluten-free bread made from roasted buckwheat flour than from raw buckwheat flour, the reverse being observed concerning FUR. Indeed, it was observed that CML and FIC were located in the negative PC1/negative compound of PC2, in the same area as the roasted buckwheat crust, but FUR was found in the area of raw buckwheat crust (Figure 1b). On the other hand, the crust and crumb were either prepared from roasted or raw buckwheat flour according to PC2, this separation being related to the higher levels of CML in the crust. It can clearly be seen that only phenolic compounds are found in the negative PC1/positive PC2 and positive PC1/positive PC2 quadrants, where the roasted and raw buckwheat bread crumb is located (Figure 1b). The phenolic compounds found in the area with the roasted and raw buckwheat breadcrumbs were 4-hydroxybenzoic acid (3), catechin (5), dihydroxybenzoic acid (6), gallic acid (8), naringenin (9), rutin (11), and *p*-coumaric acid, (14) as well as 1-O-sinapoyl-beta-D-glucose (1), ferulic acid (7), syringic acid (12), and vanillic acid (13). 

## 3. Materials and Methods

Raw and roasted buckwheat flour (Glutenex, Sady, Poland) from Polish commercial buckwheat (*Fagopyrum esculentum* Moench) were purchased from a local market. The producer confirmed that the carbohydrate, protein, and fat contents in the raw and roasted flour were: 63 and 69 g/100g, 13 and 12 g/100g, and 3.1 and 2.9 g/100g, respectively.

### 3.1. Bread-Making Process

Samples of bread were prepared using 200 g of raw or roasted buckwheat flour, 3 g of salt, 18 g of sugar, 20 g of oil, 3 g of yeast, and 160 g of water, following the recipe of Pongjaruvat et al. [39]. All the ingredients were mixed together using a KitchenAid mixer (KitchenAid, Benton Harbor, MI, USA) for 8 min at a speed of 70 rpm. After 60 min of fermentation (at 35 °C, with relative humidity 75%), the dough was divided into two parts of equal weight, placed into baking forms, and proofed for 20 min. The dough was then baked at 230 °C for 35 min in an oven (MIWE Michael Wenz, Amstein, Germany) on a semitechnical scale. Afterward, the buckwheat bread was left at room temperature for 2 h to cool down and then sliced (about 1.5 cm thick). The crust and crumb were carefully separated and ground for later analysis.

### 3.2. Extraction and Analysis of Polyphenolic Compounds

A mixture of methanol, water, and formic acid (70:29.7:0.3 *v*/*v*/*v*) was used to extract phenolic compounds from the buckwheat bread crumb and crust [40]. The compounds present in each sample were identified based on the retention time of standard and molecular mass and structural information from the MS detector during MS/MS experiments. Limit of quantification (LOQ where S/N > 15) was determined for hydroxybenzoic acid, caffeic acid, catechin, dihydroxybenzoic acid, ferulic acid, gallic acid, naringenin, quercetin; syringic acid, vanillic acid, *p*-coumaric acid and it was not lower than 0.01 µg/mL; LOQ for rutin was 0.05 µg/mL. Sinapoyl glucoside and hydroxybenzoate glucoside were quantified as sinapic acid and 4-hydroxybenzoic acid, respectively. Calibration and quality control (QC) samples were prepared in methanol, water, and formic acid (70:29.7:0.3 *v*/*v*/*v*). Recovery of QC was higher than 93% and recovery of standards spiked to extracts of buckwheat bread crumb and crust samples was in the range 86–110%. Carryover between injections was not observed. The coefficient of determination (R^2^) for all calibration curves was higher than 0.995.

Analysis was performed using reversed-phase (C18 column) ultra-high performance liquid chromatography–electrospray ionization mass spectrometry (RP-UHPLC-ESI-MS, Dionex UltiMate 3000 UHPLC; Thermo Fisher Scientific, Sunnyvale, CA, USA), following the method described by Dziedzic et al. [41].

### 3.3. Antioxidant Activity

The same extracts obtained for the determination of polyphenolic compounds were used for the analysis of antioxidant activity. One gram of lyophilized and ground sample was extracted in 7 mL of an aqueous solution with 70% methanol, 29.7% water, and 0.3% formic acid and the extraction was carried out in a water bath at 70 °C for 45 min with mechanical shaking. The total antioxidant activity was measured using the ABTS radical (2,20-azinobis-(3-ethylbenzothiazoline-6-sulfonic acid)) following the method described by Re et al. [42]. The results were expressed in µmols Trolox Equivalent Antioxidant Capacity (TEAC) per 1 g of dry matter of bread sample.

### 3.4. Analysis of Maillard Reaction Products

Furosine (2-furoylmethyl-lysine) was determined using the method described by Gökmen et al. [28]. The hydrolyzed sample was filtered through medium-grade filter paper and the diluted filtrate was passed through an Oasis HLB cartridge to remove any dark-colored interfering compounds before analysis with HPLC. A Waters Alliance HPLC System 600 (Milord, MA, USA) with a photodiode array detector was used. The furosine quantitation was based on a calibration curve by the external standard method (limit of detection (LOD) 5.75 ng/mL; LOQ 11.93 ng/mL). The results were expressed as mg/kg of sample.

Free fluorescent intermediate compounds (FIC) were determined as described by Delgado-Andrade et al. [43] and measured at λ Ex = 340 and λ Em = 420 nm using a fluorescence spectrophotometer (Shimadzu RF5001 PC, City, Japan). FIC data were expressed as mean values in fluorescence intensity (FI)/g sample.

CML (Nɛ (carboxymethyl)-l-lysine) was determined following the method described by Mildner-Szkudlarz et al. [25]. Following defatting, protein reduction, hydrolysis, and derivatization using *o*-phthaldialdehyde; CML determination was performed using a Waters Alliance HPLC system 600 (Milord, City, MA, USA) with a fluorescence detector (Waters 474). The compounds identified were quantified using the external standard calibration procedure. The limit of detection (LOD) was 0.42 ng, and the limit of quantification (LOQ) was 1.29 ng. The results were expressed as mg/kg of sample.

### 3.5. Statistical Analysis

All analyses were carried out in triplicate (*n* = 3) for each sample. Tukey’s honest significant difference multiple comparison (one-way ANOVA) at a *p* < 0.05 level was performed and Pearson correlations were determined using Statistica 13 software (Dell Software, city, state abbr. USA). Principal component analysis (PCA) was performed using selected data from the analysis. The results are presented as a two-dimensional system (biplot) obtained by plotting the observations and variables on the plane formed of the calculated principal components.

## 4. Conclusions

This is the first study reporting the formation of Maillard reaction products in buckwheat bread produced using raw and roasted flour. Buckwheat bread-making not only affects the formation of compounds with antioxidant activity but also promotes potentially harmful Maillard reaction products including FUR, FIC, and CML. However, the results suggest that raw buckwheat flour is a more appropriate ingredient for the production of buckwheat bread because the application of roasted buckwheat flour led to a higher concentration of fluorescent intermediate compounds and also the advanced glycation products formation. Taking into account the possibility of reducing the formation of potentially toxic MRPs, further investigation, especially on the role of dicarbonyl compounds, is required to better understand the mechanism of the Maillard reaction in this type of food matrix.

## Figures and Tables

**Table 1:** Phenolic compounds and antioxidant activity of raw and roasted buckwheat bread.

(µg/g)	Raw Buckwheat Bread		Roasted Buckwheat Bread	
	Crumb	Crust	Crumb	Crust
1-*O*-Sinapoyl-beta-d-glucose	nd	0.19 ± 0.01 ^b^	0.25 ± 0.03 ^a,b^	0.26 ± 0.002 ^a^
4-Hydroxybenzoate-*O*-glucoside	4.97 ± 0.47 ^a^	3.26 ± 0.06 ^b^	nd	nd
4-Hydroxybenzoic acid	0.73 ± 0.02 ^b^	0.78 ± 0.01 ^b^	9.43 ± 0.18 ^a^	8.70 ± 0.45 ^a^
Caffeic acid	0.48 ± 0.01 ^a^	0.38 ± 0.03 ^b^	0.12 ± 0.01 ^c^	0.070 ± 0.009 ^c^
Catechin	3.98 ± 0.01 ^b^	3.60 ± 0.33 ^b^	22.08 ± 1.08 ^a^	20.12 ± 0.63 ^a^
Dihydroxybenzoic acid	nd	nd	24.6 ± 1.03 ^a^	22.27 ± 1.37 ^a^
Ferulic acid	nd	nd	0.15 ± 0.009^a^	0.17 ± 0.001^a^
Gallic acid	1.47 ± 0.13 ^b^	1.68 ± 0.05 ^b^	25.46 ± 0.08 ^a^	23.61 ± 1.07 ^a^
Naringenin	nd	nd	0.09 ± 0.01 ^a^	0.07 ± 0.01^a^
Quercetin	40.42 ± 0.89 ^a^	41.75 ± 1.7 ^a^	0.96 ± 0.11 ^b^	0.9 ± 0.014 ^b^
Quercetin 3β-d-rutinoside (rutin)	nd	nd	62.52 ± 0.9 ^a^	52.41 ± 1.25 ^b^
Syringic acid	2.12 ± 0.05 ^b^	2.25 ± 0.09 ^b^	7.77 ± 0.22 ^a^	8.41 ± 0.22 ^a^
Vanillic acid	nd	nd	2.26 ± 0.15 ^a^	2.5 ± 0.23 ^a^
*p*-coumaric acid	0.26 ± 0.03 ^b^	0.23 ± 0.07 ^b^	0.38 ± 0.015 ^a^	0.370 ± 0.012 ^a^
Total	54.43 ± 0.89 ^c^	54.12 ± 1.70 ^c^	156.07 ± 0.22 ^a^	139.86 ± 1.37 ^b^
Antioxidant activity (TEAC/g sample)	3.65 ± 0.04 ^b^	4.40 ± 0.03 ^a^	2.80 ± 0.03 ^c^	2.97 ± 0.09 ^c^

Results are the mean ± standard deviation. Different letters (a, b, c) in the same row mean significant differences (*p* < 0.05). nd—not detected.

**Table 2:** Formation of Maillard reaction products (MRPs) in buckwheat bread formulated with raw and roasted buckwheat flour.

MRPs	Raw Buckwheat Bread		Roasted Buckwheat Bread	
	Crumb	Crust	Crumb	Crust
FUR (mg/kg)	71.61 ± 5.34 ^b^	117.56 ± 5.96 ^a^	6.34 ± 0.68 ^c^	8.73 ± 1.82 ^c^
FIC (FI/g)	44.43 ± 1.08 ^b^	47.07 ± 1.29 ^b^	62.06 ± 2.17 ^a^	65.05 ± 1.15 ^a^
CML (mg/kg)	423.37 ± 10.61^c^	508.85 ± 18.61 ^b^	418.56 ± 11.4 ^c^	617.86 ± 22.18 ^a^

Results are mean ± standard deviation. Different letters (a, b, c) in the same row mean significant differences (*p* < 0.05). FUR—furosine; FIC—fluorescent intermediate compounds; CML—Nɛ-carboxymethyl lysine.

**Table 3:** Correlation coefficients between MRPs, phenolic compounds and antioxidant activity for raw and roasted buckwheat bread.

	FUR	FIC	CML	TEAC
FUR		−0.89 *	−0.16	0.99 *
FIC	−0.89 *		0.48	−0.85 *
CML	−0.16	0.48		−0.05
TEAC	0.99 *	−0.85 *	−0.05	
1-*O*-Sinapoyl-beta-d-glucose	−0.47	0.78 *	0.50	−0.41
4-Hydroxybenzoate-*O*-glucoside	0.79 *	−0.96 *	−0.41	0.76 *
4-Hydroxybenzoic acid	−0.93 *	0.97 *	0.26	−0.91 *
Caffeic acid	0.83 *	−0.99 *	−0.47	0.79 *
Catechin	−0.94 *	0.96 *	0.24	−0.92 *
Dihydroxybenzoic acid	−0.93 *	0.97 *	0.25	−0.91 *
Ferulic acid	−0.93 *	0.99 *	0.40	−0.90 *
Gallic acid	−0.93 *	0.97 *	0.27	−0.91 *
Naringenin	−0.93 *	0.95 *	0.20	−0.91 *
Quercetin	0.94 *	−0.98 *	−0.31	0.91 *
Rutin	−0.93 *	0.95 *	0.21	−0.91 *
Syringic acid	−0.93 *	0.99 *	0.39	−0.89 *
Vanillic acid	−0.93 *	0.99 *	0.38	−0.90 *
*p*-coumaric acid	−0.97 *	0.94	0.18	−0.96 *

* Correlation is significant (*p* < 0.05); n = 4; FUR—furosine; FIC—fluorescent intermediate compounds; CML—Nɛ-carboxymethyl lysine.

## Data Availability

The datasets generated during and/or analyzed during the current study are available from the corresponding author on reasonable request.

## Sample Availability

Samples of bread are available from the corresponding author.
